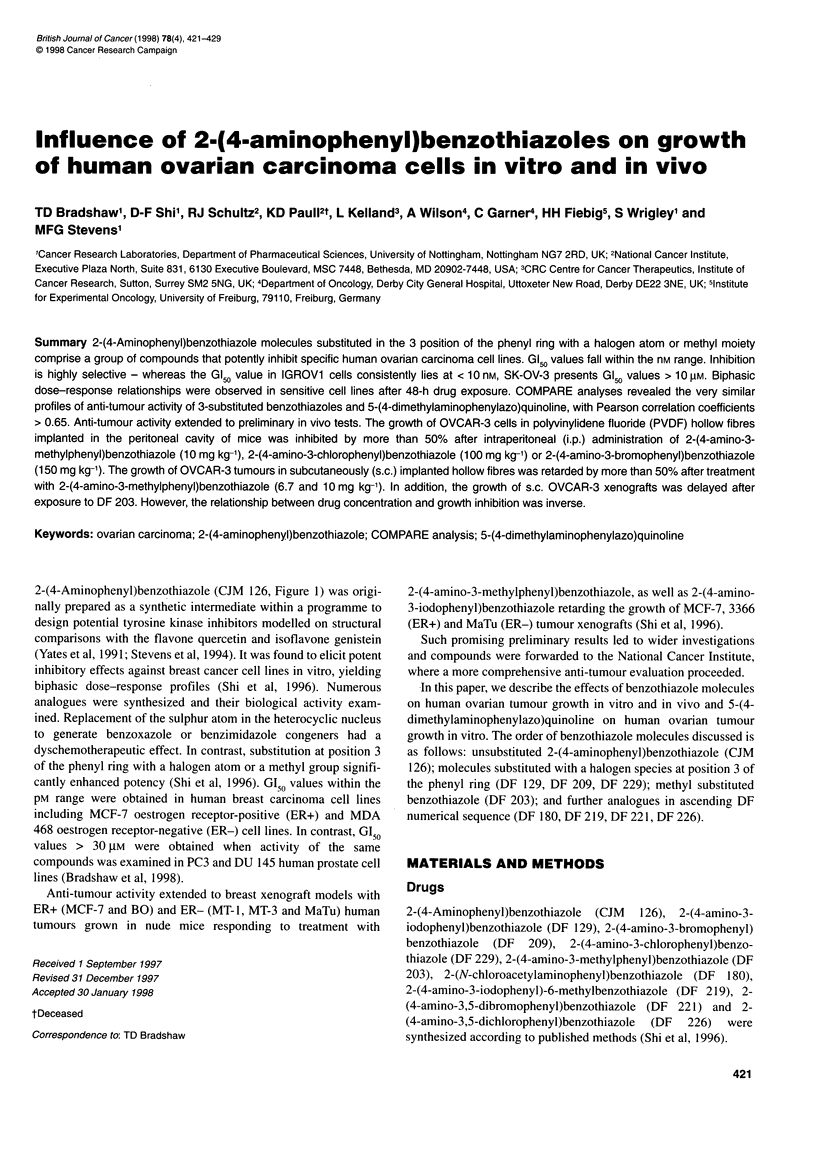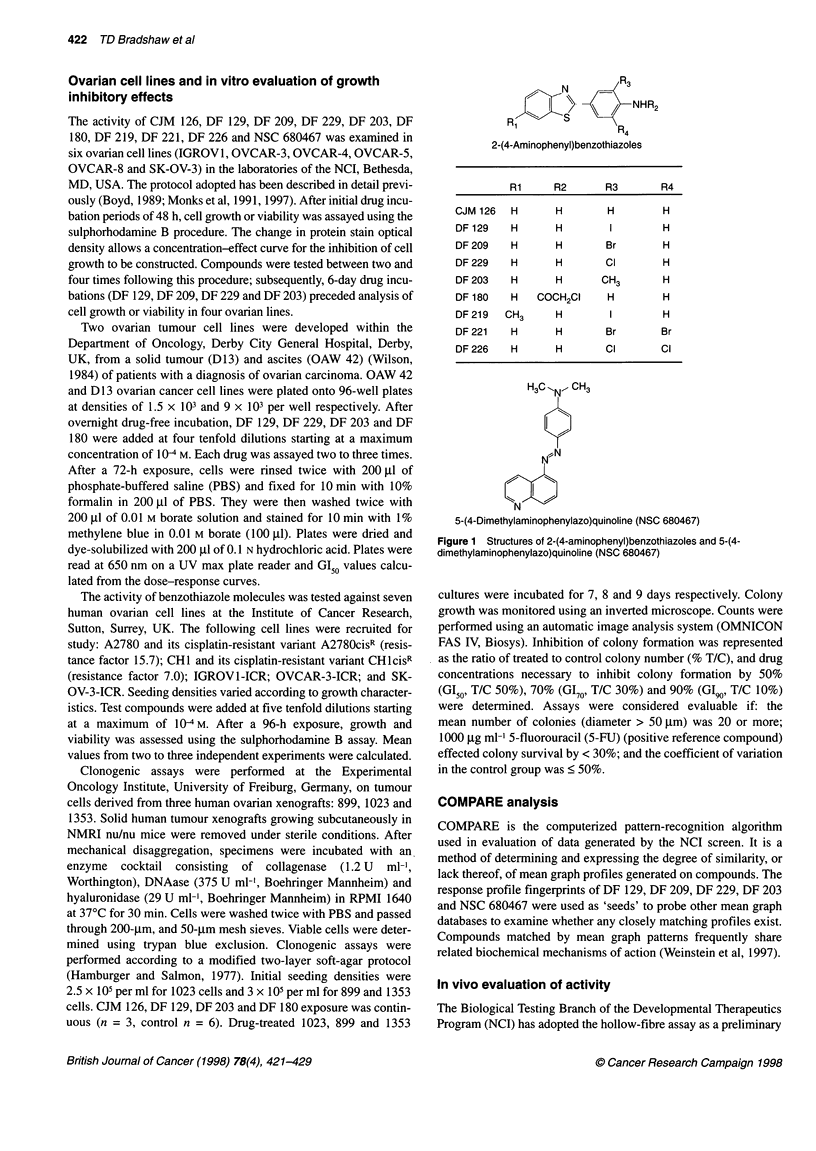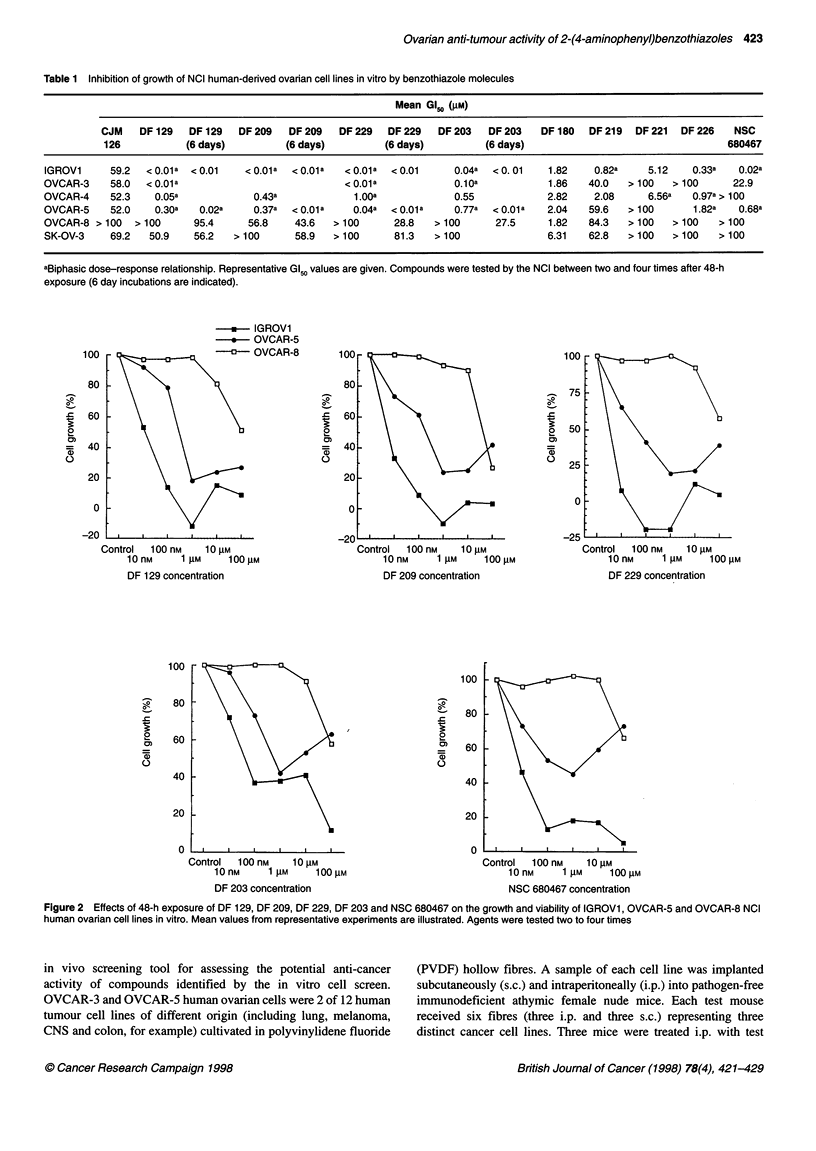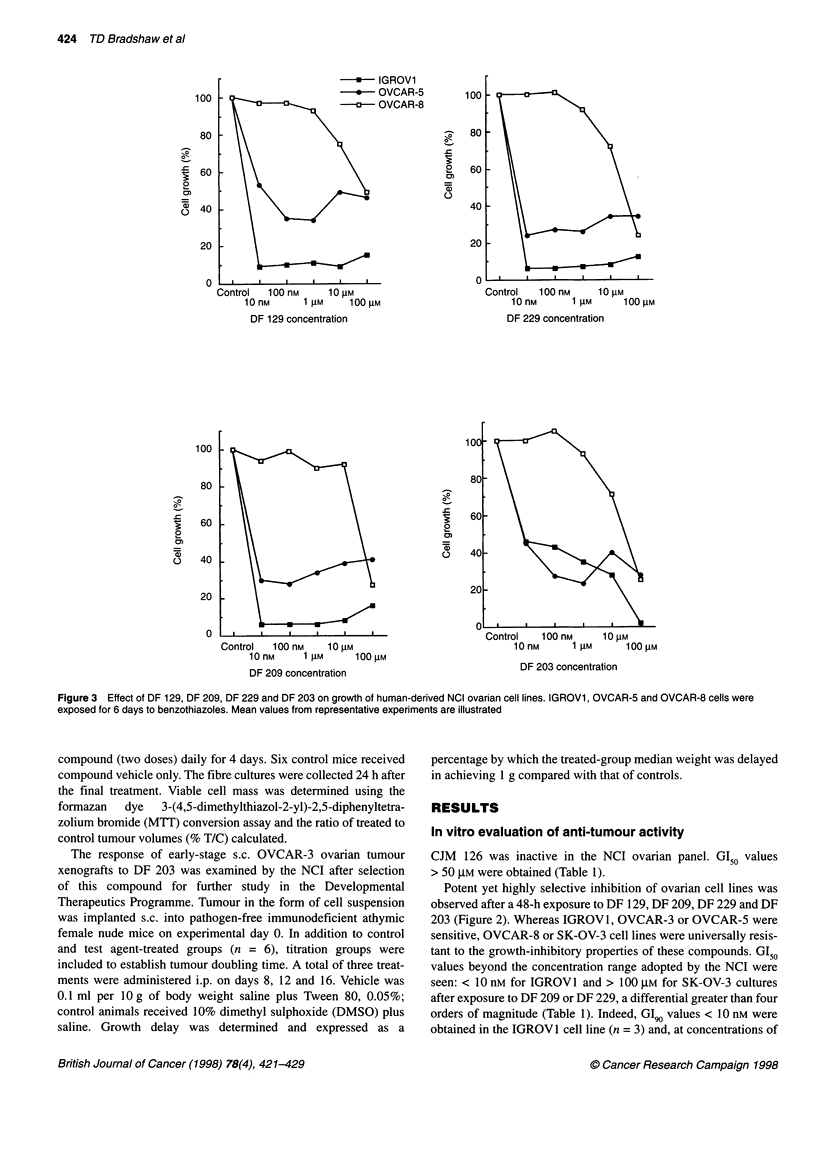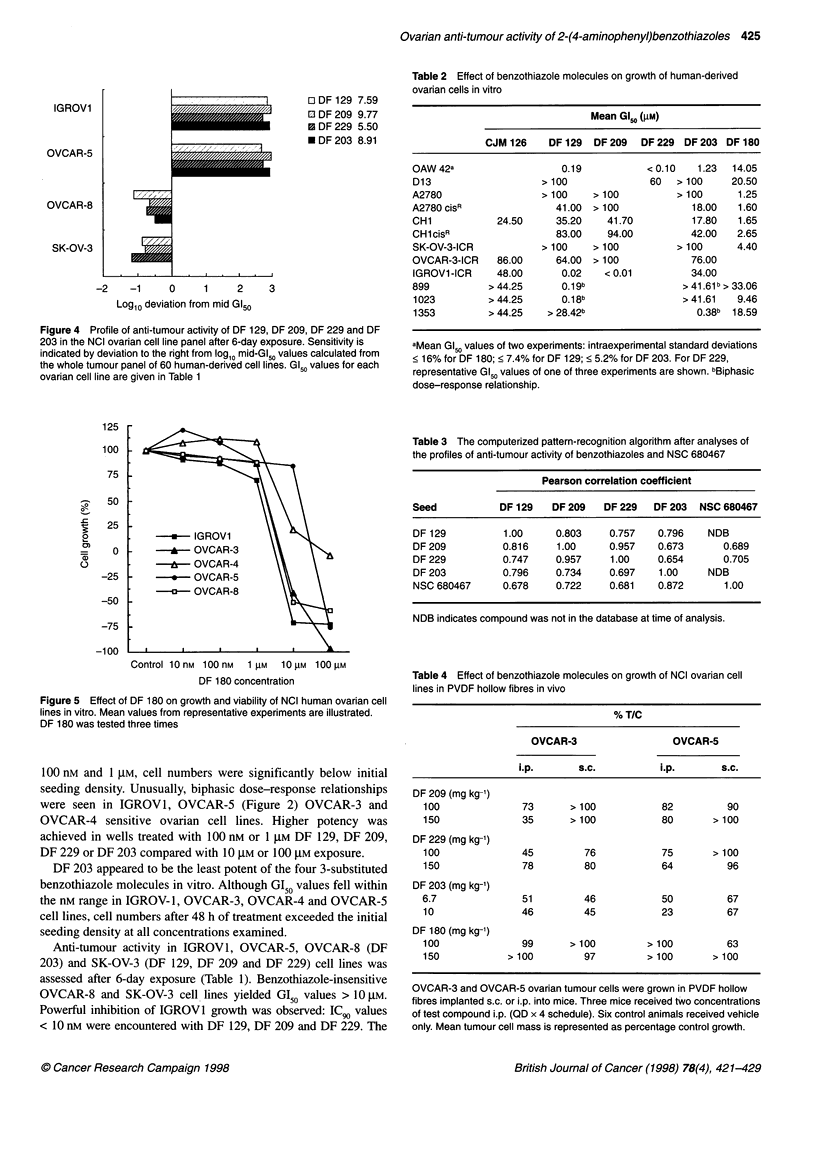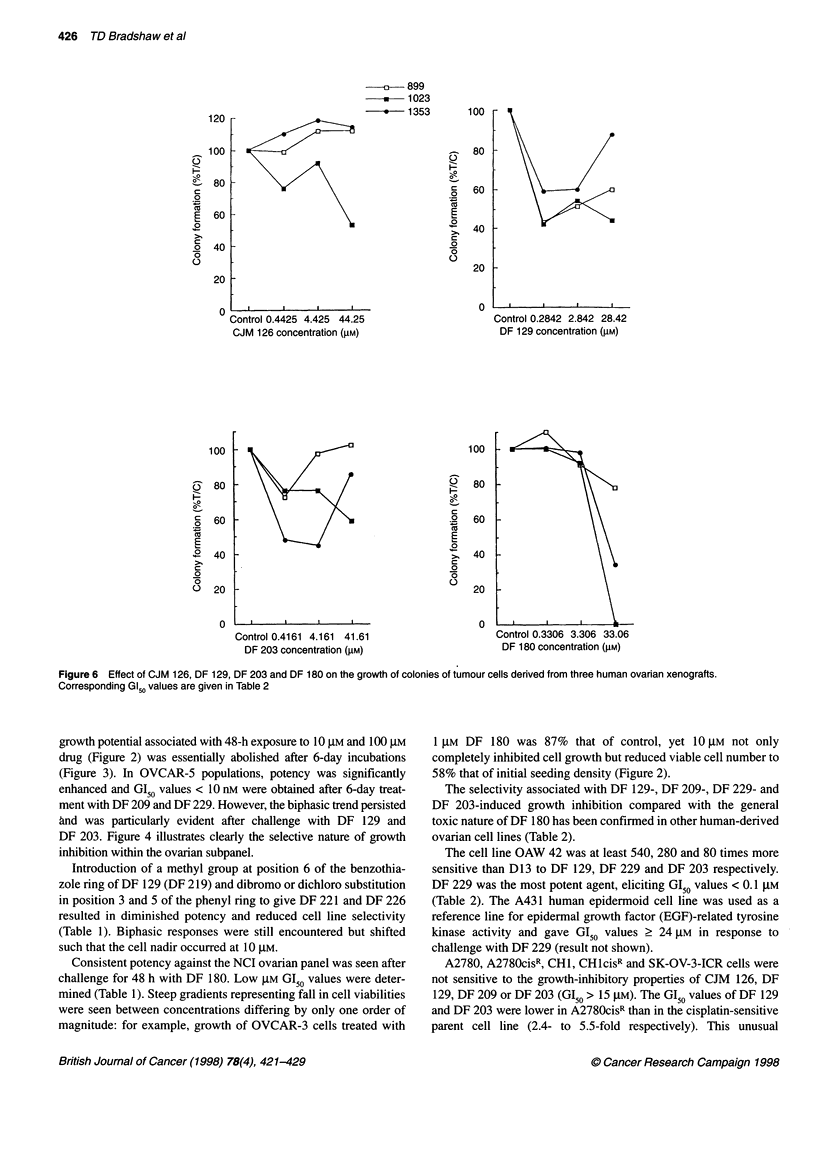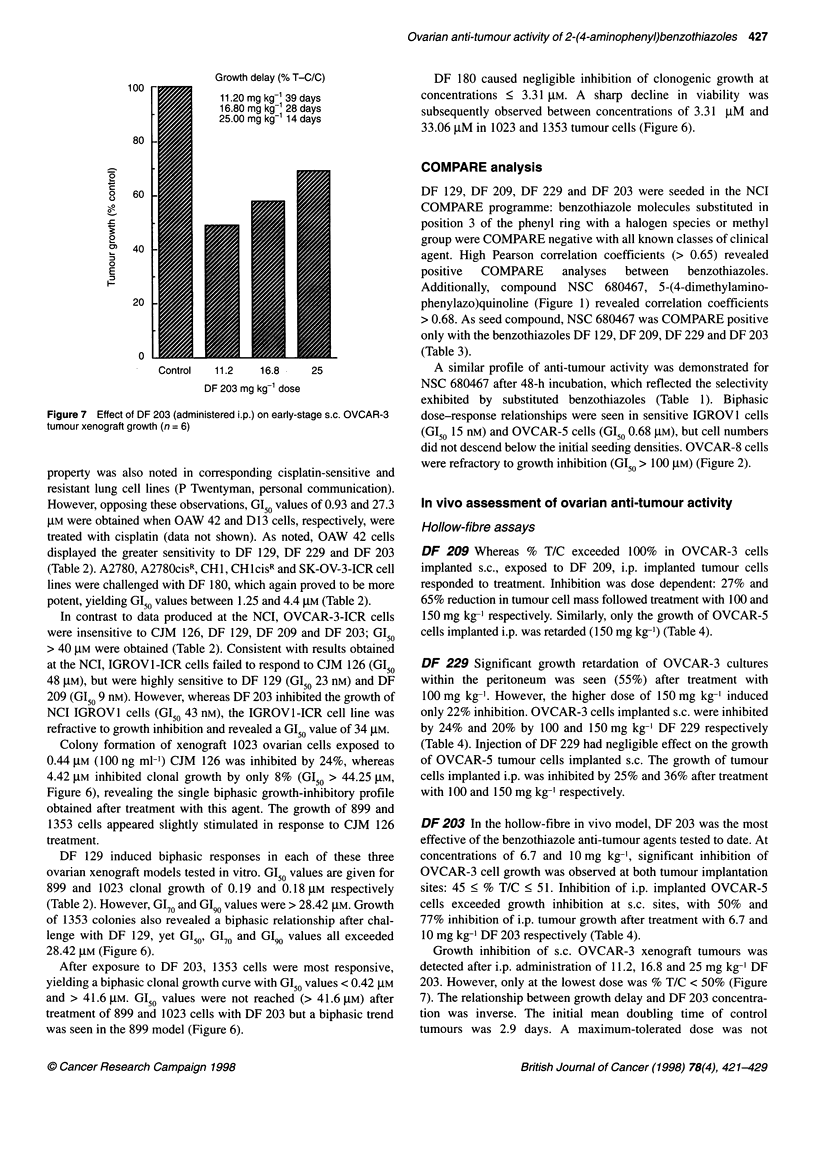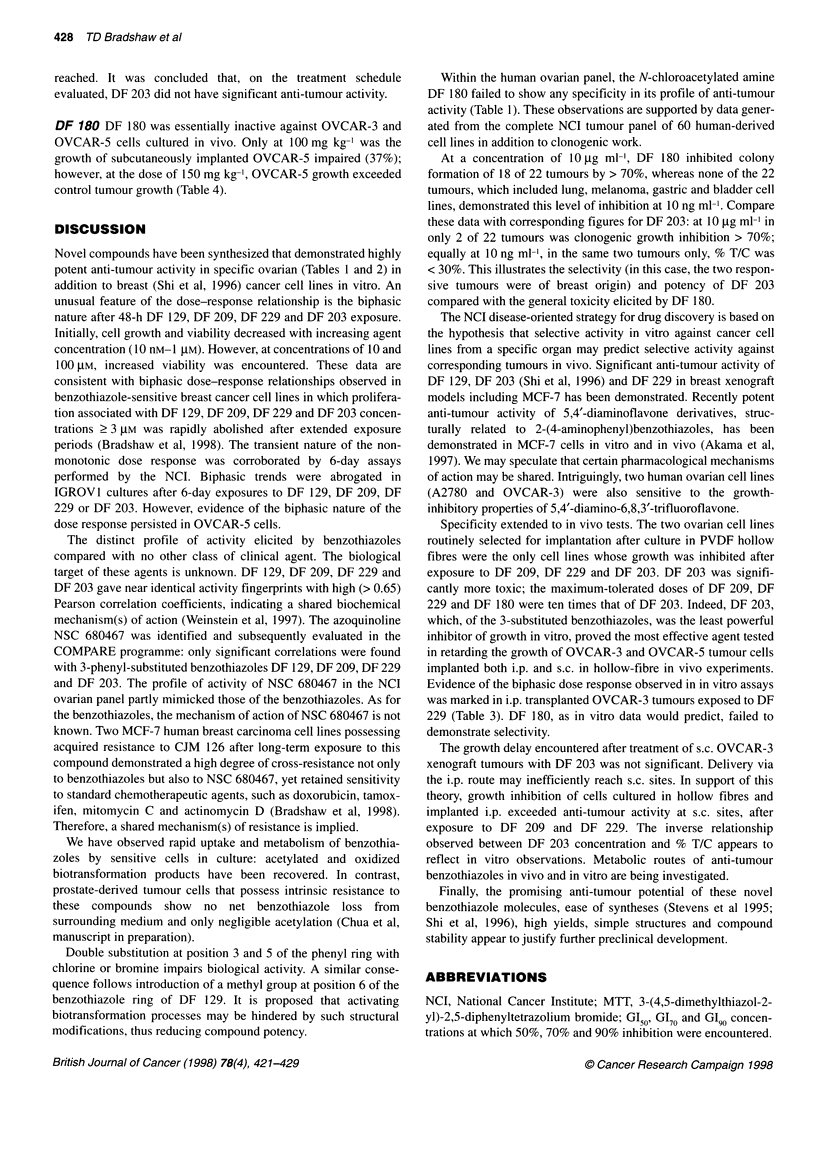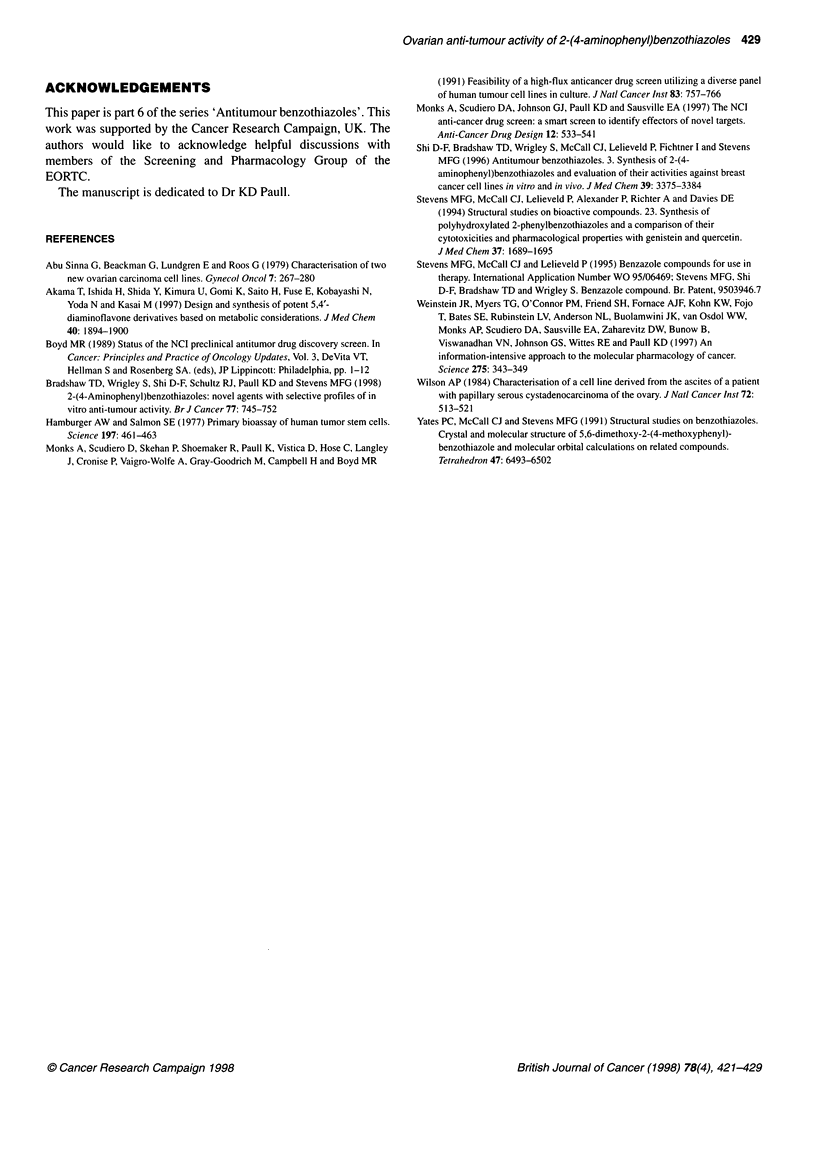# Influence of 2-(4-aminophenyl)benzothiazoles on growth of human ovarian carcinoma cells in vitro and in vivo.

**DOI:** 10.1038/bjc.1998.510

**Published:** 1998-08

**Authors:** T. D. Bradshaw, D. F. Shi, R. J. Schultz, K. D. Paull, L. Kelland, A. Wilson, C. Garner, H. H. Fiebig, S. Wrigley, M. F. Stevens

**Affiliations:** Department of Pharmaceutical Sciences, University of Nottingham, UK.

## Abstract

2-(4-Aminophenyl)benzothiazole molecules substituted in the 3 position of the phenyl ring with a halogen atom or methyl moiety comprise a group of compounds that potently inhibit specific human ovarian carcinoma cell lines. GI50 values fall within the nM range. Inhibition is highly selective -- whereas the GI50 value in IGROV1 cells consistently lies at < 10 nM, SK-OV-3 presents GI50 values > 10 microM. Biphasic dose-response relationships were observed in sensitive cell lines after 48-h drug exposure. COMPARE analyses revealed the very similar profiles of anti-tumour activity of 3-substituted benzothiazoles and 5-(4-dimethylaminophenylazo)quinoline, with Pearson correlation coefficients > 0.65. Anti-tumour activity extended to preliminary in vivo tests. The growth of OVCAR-3 cells in polyvinylidene fluoride (PVDF) hollow fibres implanted in the peritoneal cavity of mice was inhibited by more than 50% after intraperitoneal (i.p.) administration of 2-(4-amino-3-methylphenyl)benzothiazole (10 mg kg(-1)), 2-(4-amino-3-chlorophenyl)benzothiazole (100 mg kg(-1)) or 2-(4-amino-3-bromophenyl)benzothiazole (150 mg kg(-1)). The growth of OVCAR-3 tumours in subcutaneously (s.c.) implanted hollow fibres was retarded by more than 50% after treatment with 2-(4-amino-3-methylphenyl)benzothiazole (6.7 and 10 mg kg(-1)). In addition, the growth of s.c. OVCAR-3 xenografts was delayed after exposure to DF 203. However, the relationship between drug concentration and growth inhibition was inverse.


British Journal of Cancer (1998) 78(4), 421–429
© 1998 Cancer Research Campaign

### Influence of 2-(4-aminophenyl)benzothiazoles on growth of human ovarian carcinoma cells in vitro and in vivo

**TD Bradshaw[1], D-F Shi[1], RJ Schultz[2], KD Paull[2†], L Kelland[3], A Wilson[4], C Garner[4], HH Fiebig[5], S Wrigley[1] and MFG Stevens[1]**

[1]Cancer Research Laboratories, Department of Pharmaceutical Sciences, University of Nottingham, Nottingham NG7 2RD, UK; [2]National Cancer Institute, Executive Plaza North, Suite 831, 6130 Executive Boulevard, MSC 7448, Bethesda, MD 20902-7448, USA; [3]CRC Centre for Cancer Therapeutics, Institute of Cancer Research, Sutton, Surrey SM2 5NG, UK; [4]Department of Oncology, Derby City General Hospital, Uttoxeter New Road, Derby DE22 3NE, UK; [5]Institute for Experimental Oncology, University of Freiburg, 79110, Freiburg, Germany

**Summary** 2-(4-Aminophenyl)benzothiazole molecules substituted in the 3 position of the phenyl ring with a halogen atom or methyl moiety comprise a group of compounds that potently inhibit specific human ovarian carcinoma cell lines. $GI_{50}$ values fall within the nM range. Inhibition is highly selective – whereas the $GI_{50}$ value in IGROV1 cells consistently lies at < 10 nM, SK-OV-3 presents $GI_{50}$ values > 10 μM. Biphasic dose–response relationships were observed in sensitive cell lines after 48-h drug exposure. COMPARE analyses revealed the very similar profiles of anti-tumour activity of 3-substituted benzothiazoles and 5-(4-dimethylaminophenylazo)quinoline, with Pearson correlation coefficients > 0.65. Anti-tumour activity extended to preliminary in vivo tests. The growth of OVCAR-3 cells in polyvinylidene fluoride (PVDF) hollow fibres implanted in the peritoneal cavity of mice was inhibited by more than 50% after intraperitoneal (i.p.) administration of 2-(4-amino-3-methylphenyl)benzothiazole (10 mg $kg^{-1}$), 2-(4-amino-3-chlorophenyl)benzothiazole (100 mg $kg^{-1}$) or 2-(4-amino-3-bromophenyl)benzothiazole (150 mg $kg^{-1}$). The growth of OVCAR-3 tumours in subcutaneously (s.c.) implanted hollow fibres was retarded by more than 50% after treatment with 2-(4-amino-3-methylphenyl)benzothiazole (6.7 and 10 mg $kg^{-1}$). In addition, the growth of s.c. OVCAR-3 xenografts was delayed after exposure to DF 203. However, the relationship between drug concentration and growth inhibition was inverse.

**Keywords:** ovarian carcinoma; 2-(4-aminophenyl)benzothiazole; COMPARE analysis; 5-(4-dimethylaminophenylazo)quinoline

2-(4-Aminophenyl)benzothiazole (CJM 126, Figure 1) was originally prepared as a synthetic intermediate within a programme to design potential tyrosine kinase inhibitors modelled on structural comparisons with the flavone quercetin and isoflavone genistein (Yates et al, 1991; Stevens et al, 1994). It was found to elicit potent inhibitory effects against breast cancer cell lines in vitro, yielding biphasic dose–response profiles (Shi et al, 1996). Numerous analogues were synthesized and their biological activity examined. Replacement of the sulphur atom in the heterocyclic nucleus to generate benzoxazole or benzimidazole congeners had a dyschemotherapeutic effect. In contrast, substitution at position 3 of the phenyl ring with a halogen atom or a methyl group significantly enhanced potency (Shi et al, 1996). $GI_{50}$ values within the pM range were obtained in human breast carcinoma cell lines including MCF-7 oestrogen receptor-positive (ER+) and MDA 468 oestrogen receptor-negative (ER–) cell lines. In contrast, $GI_{50}$ values > 30 μM were obtained when activity of the same compounds was examined in PC3 and DU 145 human prostate cell lines (Bradshaw et al, 1998).

Anti-tumour activity extended to breast xenograft models with ER+ (MCF-7 and BO) and ER– (MT-1, MT-3 and MaTu) human tumours grown in nude mice responding to treatment with 2-(4-amino-3-methylphenyl)benzothiazole, as well as 2-(4-amino-3-iodophenyl)benzothiazole retarding the growth of MCF-7, 3366 (ER+) and MaTu (ER–) tumour xenografts (Shi et al, 1996).

Such promising preliminary results led to wider investigations and compounds were forwarded to the National Cancer Institute, where a more comprehensive anti-tumour evaluation proceeded.

In this paper, we describe the effects of benzothiazole molecules on human ovarian tumour growth in vitro and in vivo and 5-(4-dimethylaminophenylazo)quinoline on human ovarian tumour growth in vitro. The order of benzothiazole molecules discussed is as follows: unsubstituted 2-(4-aminophenyl)benzothiazole (CJM 126); molecules substituted with a halogen species at position 3 of the phenyl ring (DF 129, DF 209, DF 229); methyl substituted benzothiazole (DF 203); and further analogues in ascending DF numerical sequence (DF 180, DF 219, DF 221, DF 226).

*Received 1 September 1997*
*Revised 31 December 1997*
*Accepted 30 January 1998*

†Deceased

*Correspondence to*: TD Bradshaw

### MATERIALS AND METHODS

### Drugs

2-(4-Aminophenyl)benzothiazole (CJM 126), 2-(4-amino-3-iodophenyl)benzothiazole (DF 129), 2-(4-amino-3-bromophenyl) benzothiazole (DF 209), 2-(4-amino-3-chlorophenyl)benzothiazole (DF 229), 2-(4-amino-3-methylphenyl)benzothiazole (DF 203), 2-(*N*-chloroacetylaminophenyl)benzothiazole (DF 180), 2-(4-amino-3-iodophenyl)-6-methylbenzothiazole (DF 219), 2-(4-amino-3,5-dibromophenyl)benzothiazole (DF 221) and 2-(4-amino-3,5-dichlorophenyl)benzothiazole (DF 226) were synthesized according to published methods (Shi et al, 1996).

2-(4-Aminophenyl)benzothiazoles

| | R1 | R2 | R3 | R4 |
|---|---|---|---|---|
| CJM 126 | H | H | H | H |
| DF 129 | H | H | I | H |
| DF 209 | H | H | Br | H |
| DF 229 | H | H | Cl | H |
| DF 203 | H | H | $CH_3$ | H |
| DF 180 | H | $COCH_2Cl$ | H | H |
| DF 219 | $CH_3$ | H | I | H |
| DF 221 | H | H | Br | Br |
| DF 226 | H | H | Cl | Cl |

5-(4-Dimethylaminophenylazo)quinoline (NSC 680467)

**Figure 1** Structures of 2-(4-aminophenyl)benzothiazoles and 5-(4-dimethylaminophenylazo)quinoline (NSC 680467)

### Ovarian cell lines and in vitro evaluation of growth inhibitory effects

The activity of CJM 126, DF 129, DF 209, DF 229, DF 203, DF 180, DF 219, DF 221, DF 226 and NSC 680467 was examined in six ovarian cell lines (IGROV1, OVCAR-3, OVCAR-4, OVCAR-5, OVCAR-8 and SK-OV-3) in the laboratories of the NCI, Bethesda, MD, USA. The protocol adopted has been described in detail previously (Boyd, 1989; Monks et al, 1991, 1997). After initial drug incubation periods of 48 h, cell growth or viability was assayed using the sulphorhodamine B procedure. The change in protein stain optical density allows a concentration–effect curve for the inhibition of cell growth to be constructed. Compounds were tested between two and four times following this procedure; subsequently, 6-day drug incubations (DF 129, DF 209, DF 229 and DF 203) preceded analysis of cell growth or viability in four ovarian lines.

Two ovarian tumour cell lines were developed within the Department of Oncology, Derby City General Hospital, Derby, UK, from a solid tumour (D13) and ascites (OAW 42) (Wilson, 1984) of patients with a diagnosis of ovarian carcinoma. OAW 42 and D13 ovarian cancer cell lines were plated onto 96-well plates at densities of $1.5 \times 10^3$ and $9 \times 10^3$ per well respectively. After overnight drug-free incubation, DF 129, DF 229, DF 203 and DF 180 were added at four tenfold dilutions starting at a maximum concentration of $10^{-4}$ M. Each drug was assayed two to three times. After a 72-h exposure, cells were rinsed twice with 200 µl of phosphate-buffered saline (PBS) and fixed for 10 min with 10% formalin in 200 µl of PBS. They were then washed twice with 200 µl of 0.01 M borate solution and stained for 10 min with 1% methylene blue in 0.01 M borate (100 µl). Plates were dried and dye-solubilized with 200 µl of 0.1 N hydrochloric acid. Plates were read at 650 nm on a UV max plate reader and $GI_{50}$ values calculated from the dose–response curves.

The activity of benzothiazole molecules was tested against seven human ovarian cell lines at the Institute of Cancer Research, Sutton, Surrey, UK. The following cell lines were recruited for study: A2780 and its cisplatin-resistant variant A2780cis$^R$ (resistance factor 15.7); CH1 and its cisplatin-resistant variant CH1cis$^R$ (resistance factor 7.0); IGROV1-ICR; OVCAR-3-ICR; and SK-OV-3-ICR. Seeding densities varied according to growth characteristics. Test compounds were added at five tenfold dilutions starting at a maximum of $10^{-4}$ M. After a 96-h exposure, growth and viability was assessed using the sulphorhodamine B assay. Mean values from two to three independent experiments were calculated.

Clonogenic assays were performed at the Experimental Oncology Institute, University of Freiburg, Germany, on tumour cells derived from three human ovarian xenografts: 899, 1023 and 1353. Solid human tumour xenografts growing subcutaneously in NMRI nu/nu mice were removed under sterile conditions. After mechanical disaggregation, specimens were incubated with an enzyme cocktail consisting of collagenase (1.2 U ml$^{-1}$, Worthington), DNAase (375 U ml$^{-1}$, Boehringer Mannheim) and hyaluronidase (29 U ml$^{-1}$, Boehringer Mannheim) in RPMI 1640 at 37°C for 30 min. Cells were washed twice with PBS and passed through 200-µm, and 50-µm mesh sieves. Viable cells were determined using trypan blue exclusion. Clonogenic assays were performed according to a modified two-layer soft-agar protocol (Hamburger and Salmon, 1977). Initial seeding densities were $2.5 \times 10^5$ per ml for 1023 cells and $3 \times 10^5$ per ml for 899 and 1353 cells. CJM 126, DF 129, DF 203 and DF 180 exposure was continuous ($n$ = 3, control $n$ = 6). Drug-treated 1023, 899 and 1353 cultures were incubated for 7, 8 and 9 days respectively. Colony growth was monitored using an inverted microscope. Counts were performed using an automatic image analysis system (OMNICON FAS IV, Biosys). Inhibition of colony formation was represented as the ratio of treated to control colony number (% T/C), and drug concentrations necessary to inhibit colony formation by 50% ($GI_{50}$, T/C 50%), 70% ($GI_{70}$, T/C 30%) and 90% ($GI_{90}$, T/C 10%) were determined. Assays were considered evaluable if: the mean number of colonies (diameter > 50 µm) was 20 or more; 1000 µg ml$^{-1}$ 5-fluorouracil (5-FU) (positive reference compound) effected colony survival by < 30%; and the coefficient of variation in the control group was ≤ 50%.

### COMPARE analysis

COMPARE is the computerized pattern-recognition algorithm used in evaluation of data generated by the NCI screen. It is a method of determining and expressing the degree of similarity, or lack thereof, of mean graph profiles generated on compounds. The response profile fingerprints of DF 129, DF 209, DF 229, DF 203 and NSC 680467 were used as 'seeds' to probe other mean graph databases to examine whether any closely matching profiles exist. Compounds matched by mean graph patterns frequently share related biochemical mechanisms of action (Weinstein et al, 1997).

### In vivo evaluation of activity

The Biological Testing Branch of the Developmental Therapeutics Program (NCI) has adopted the hollow-fibre assay as a preliminary

© Cancer Research Campaign 1998

**Table 1** Inhibition of growth of NCI human-derived ovarian cell lines in vitro by benzothiazole molecules

| | Mean $GI_{50}$ (μM) | | | | | | | | | | | | | |
|---|---|---|---|---|---|---|---|---|---|---|---|---|---|---|
| | **CJM 126** | **DF 129** | **DF 129 (6 days)** | **DF 209** | **DF 209 (6 days)** | **DF 229** | **DF 229 (6 days)** | **DF 203** | **DF 203 (6 days)** | **DF 180** | **DF 219** | **DF 221** | **DF 226** | **NSC 680467** |
| IGROV1 | 59.2 | < 0.01[a] | < 0.01 | < 0.01[a] | < 0.01[a] | < 0.01[a] | < 0.01 | 0.04[a] | < 0. 01 | 1.82 | 0.82[a] | 5.12 | 0.33[a] | 0.02[a] |
| OVCAR-3 | 58.0 | < 0.01[a] | | | | < 0.01[a] | | 0.10[a] | | 1.86 | 40.0 | > 100 | > 100 | 22.9 |
| OVCAR-4 | 52.3 | 0.05[a] | | 0.43[a] | | 1.00[a] | | 0.55 | | 2.82 | 2.08 | 6.56[a] | 0.97[a] | > 100 |
| OVCAR-5 | 52.0 | 0.30[a] | 0.02[a] | 0.37[a] | < 0.01[a] | 0.04[a] | < 0.01[a] | 0.77[a] | < 0.01[a] | 2.04 | 59.6 | > 100 | 1.82[a] | 0.68[a] |
| OVCAR-8 | > 100 | > 100 | 95.4 | 56.8 | 43.6 | > 100 | 28.8 | > 100 | 27.5 | 1.82 | 84.3 | > 100 | > 100 | > 100 |
| SK-OV-3 | 69.2 | 50.9 | 56.2 | > 100 | 58.9 | > 100 | 81.3 | > 100 | | 6.31 | 62.8 | > 100 | > 100 | > 100 |

[a]Biphasic dose–response relationship. Representative $GI_{50}$ values are given. Compounds were tested by the NCI between two and four times after 48-h exposure (6 day incubations are indicated).

**Figure 2** Effects of 48-h exposure of DF 129, DF 209, DF 229, DF 203 and NSC 680467 on the growth and viability of IGROV1, OVCAR-5 and OVCAR-8 NCI human ovarian cell lines in vitro. Mean values from representative experiments are illustrated. Agents were tested two to four times in vivo screening tool for assessing the potential anti-cancer activity of compounds identified by the in vitro cell screen. OVCAR-3 and OVCAR-5 human ovarian cells were 2 of 12 human tumour cell lines of different origin (including lung, melanoma, CNS and colon, for example) cultivated in polyvinylidene fluoride (PVDF) hollow fibres. A sample of each cell line was implanted subcutaneously (s.c.) and intraperitoneally (i.p.) into pathogen-free immunodeficient athymic female nude mice. Each test mouse received six fibres (three i.p. and three s.c.) representing three distinct cancer cell lines. Three mice were treated i.p. with test

© Cancer Research Campaign 1998

**Figure 3** Effect of DF 129, DF 209, DF 229 and DF 203 on growth of human-derived NCI ovarian cell lines. IGROV1, OVCAR-5 and OVCAR-8 cells were exposed for 6 days to benzothiazoles. Mean values from representative experiments are illustrated compound (two doses) daily for 4 days. Six control mice received compound vehicle only. The fibre cultures were collected 24 h after the final treatment. Viable cell mass was determined using the formazan dye 3-(4,5-dimethylthiazol-2-yl)-2,5-diphenyltetrazolium bromide (MTT) conversion assay and the ratio of treated to control tumour volumes (% T/C) calculated.

The response of early-stage s.c. OVCAR-3 ovarian tumour xenografts to DF 203 was examined by the NCI after selection of this compound for further study in the Developmental Therapeutics Programme. Tumour in the form of cell suspension was implanted s.c. into pathogen-free immunodeficient athymic female nude mice on experimental day 0. In addition to control and test agent-treated groups ($n$ = 6), titration groups were included to establish tumour doubling time. A total of three treatments were administered i.p. on days 8, 12 and 16. Vehicle was 0.1 ml per 10 g of body weight saline plus Tween 80, 0.05%; control animals received 10% dimethyl sulphoxide (DMSO) plus saline. Growth delay was determined and expressed as a percentage by which the treated-group median weight was delayed in achieving 1 g compared with that of controls.

### RESULTS

### In vitro evaluation of anti-tumour activity

CJM 126 was inactive in the NCI ovarian panel. $GI_{50}$ values > 50 μM were obtained (Table 1).

Potent yet highly selective inhibition of ovarian cell lines was observed after a 48-h exposure to DF 129, DF 209, DF 229 and DF 203 (Figure 2). Whereas IGROV1, OVCAR-3 or OVCAR-5 were sensitive, OVCAR-8 or SK-OV-3 cell lines were universally resistant to the growth-inhibitory properties of these compounds. $GI_{50}$ values beyond the concentration range adopted by the NCI were seen: < 10 nM for IGROV1 and > 100 μM for SK-OV-3 cultures after exposure to DF 209 or DF 229, a differential greater than four orders of magnitude (Table 1). Indeed, $GI_{90}$ values < 10 nM were obtained in the IGROV1 cell line ($n$ = 3) and, at concentrations of

© Cancer Research Campaign 1998

**Figure 4** Profile of anti-tumour activity of DF 129, DF 209, DF 229 and DF 203 in the NCI ovarian cell line panel after 6-day exposure. Sensitivity is indicated by deviation to the right from $\log_{10}$ mid-$GI_{50}$ values calculated from the whole tumour panel of 60 human-derived cell lines. $GI_{50}$ values for each ovarian cell line are given in Table 1

**Figure 5** Effect of DF 180 on growth and viability of NCI human ovarian cell lines in vitro. Mean values from representative experiments are illustrated. DF 180 was tested three times

100 nM and 1 µM, cell numbers were significantly below initial seeding density. Unusually, biphasic dose–response relationships were seen in IGROV1, OVCAR-5 (Figure 2) OVCAR-3 and OVCAR-4 sensitive ovarian cell lines. Higher potency was achieved in wells treated with 100 nM or 1 µM DF 129, DF 209, DF 229 or DF 203 compared with 10 µM or 100 µM exposure.

DF 203 appeared to be the least potent of the four 3-substituted benzothiazole molecules in vitro. Although $GI_{50}$ values fell within the nM range in IGROV-1, OVCAR-3, OVCAR-4 and OVCAR-5 cell lines, cell numbers after 48 h of treatment exceeded the initial seeding density at all concentrations examined.

Anti-tumour activity in IGROV1, OVCAR-5, OVCAR-8 (DF 203) and SK-OV-3 (DF 129, DF 209 and DF 229) cell lines was assessed after 6-day exposure (Table 1). Benzothiazole-insensitive OVCAR-8 and SK-OV-3 cell lines yielded $GI_{50}$ values > 10 µM. Powerful inhibition of IGROV1 growth was observed: $IC_{90}$ values < 10 nM were encountered with DF 129, DF 209 and DF 229. The

**Table 2** Effect of benzothiazole molecules on growth of human-derived ovarian cells in vitro

| | Mean $GI_{50}$ (µM) | | | | | |
|---|---|---|---|---|---|---|
| | CJM 126 | DF 129 | DF 209 | DF 229 | DF 203 | DF 180 |
| OAW 42[a] | | 0.19 | | < 0.10 | 1.23 | 14.05 |
| D13 | | > 100 | | 60 | > 100 | 20.50 |
| A2780 | | > 100 | > 100 | | > 100 | 1.25 |
| A2780 cis[R] | | 41.00 | > 100 | | 18.00 | 1.60 |
| CH1 | 24.50 | 35.20 | 41.70 | | 17.80 | 1.65 |
| CH1cis[R] | | 83.00 | 94.00 | | 42.00 | 2.65 |
| SK-OV-3-ICR | | > 100 | > 100 | | > 100 | 4.40 |
| OVCAR-3-ICR | 86.00 | 64.00 | > 100 | | 76.00 | |
| IGROV1-ICR | 48.00 | 0.02 | < 0.01 | | 34.00 | |
| 899 | > 44.25 | 0.19[b] | | | > 41.61[b] | > 33.06 |
| 1023 | > 44.25 | 0.18[b] | | | > 41.61 | 9.46 |
| 1353 | > 44.25 | > 28.42[b] | | | 0.38[b] | 18.59 |

[a]Mean $GI_{50}$ values of two experiments: intraexperimental standard deviations ≤ 16% for DF 180; ≤ 7.4% for DF 129; ≤ 5.2% for DF 203. For DF 229, representative $GI_{50}$ values of one of three experiments are shown. [b]Biphasic dose–response relationship.

**Table 3** The computerized pattern-recognition algorithm after analyses of the profiles of anti-tumour activity of benzothiazoles and NSC 680467

| | Pearson correlation coefficient | | | | |
|---|---|---|---|---|---|
| Seed | DF 129 | DF 209 | DF 229 | DF 203 | NSC 680467 |
| DF 129 | 1.00 | 0.803 | 0.757 | 0.796 | NDB |
| DF 209 | 0.816 | 1.00 | 0.957 | 0.673 | 0.689 |
| DF 229 | 0.747 | 0.957 | 1.00 | 0.654 | 0.705 |
| DF 203 | 0.796 | 0.734 | 0.697 | 1.00 | NDB |
| NSC 680467 | 0.678 | 0.722 | 0.681 | 0.872 | 1.00 |

NDB indicates compound was not in the database at time of analysis.

**Table 4** Effect of benzothiazole molecules on growth of NCI ovarian cell lines in PVDF hollow fibres in vivo

| | % T/C | | | |
|---|---|---|---|---|
| | OVCAR-3 | | OVCAR-5 | |
| | i.p. | s.c. | i.p. | s.c. |
| DF 209 (mg kg⁻¹) | | | | |
| 100 | 73 | > 100 | 82 | 90 |
| 150 | 35 | > 100 | 80 | > 100 |
| DF 229 (mg kg⁻¹) | | | | |
| 100 | 45 | 76 | 75 | > 100 |
| 150 | 78 | 80 | 64 | 96 |
| DF 203 (mg kg⁻¹) | | | | |
| 6.7 | 51 | 46 | 50 | 67 |
| 10 | 46 | 45 | 23 | 67 |
| DF 180 (mg kg⁻¹) | | | | |
| 100 | 99 | > 100 | > 100 | 63 |
| 150 | > 100 | 97 | > 100 | > 100 |

OVCAR-3 and OVCAR-5 ovarian tumour cells were grown in PVDF hollow fibres implanted s.c. or i.p. into mice. Three mice received two concentrations of test compound i.p. (QD × 4 schedule). Six control animals received vehicle only. Mean tumour cell mass is represented as percentage control growth.

© Cancer Research Campaign 1998

**Figure 6** Effect of CJM 126, DF 129, DF 203 and DF 180 on the growth of colonies of tumour cells derived from three human ovarian xenografts. Corresponding $GI_{50}$ values are given in Table 2

growth potential associated with 48-h exposure to 10 μM and 100 μM drug (Figure 2) was essentially abolished after 6-day incubations (Figure 3). In OVCAR-5 populations, potency was significantly enhanced and $GI_{50}$ values < 10 nM were obtained after 6-day treatment with DF 209 and DF 229. However, the biphasic trend persisted and was particularly evident after challenge with DF 129 and DF 203. Figure 4 illustrates clearly the selective nature of growth inhibition within the ovarian subpanel.

Introduction of a methyl group at position 6 of the benzothiazole ring of DF 129 (DF 219) and dibromo or dichloro substitution in position 3 and 5 of the phenyl ring to give DF 221 and DF 226 resulted in diminished potency and reduced cell line selectivity (Table 1). Biphasic responses were still encountered but shifted such that the cell nadir occurred at 10 μM.

Consistent potency against the NCI ovarian panel was seen after challenge for 48 h with DF 180. Low μM $GI_{50}$ values were determined (Table 1). Steep gradients representing fall in cell viabilities were seen between concentrations differing by only one order of magnitude: for example, growth of OVCAR-3 cells treated with 1 μM DF 180 was 87% that of control, yet 10 μM not only completely inhibited cell growth but reduced viable cell number to 58% that of initial seeding density (Figure 2).

The selectivity associated with DF 129-, DF 209-, DF 229- and DF 203-induced growth inhibition compared with the general toxic nature of DF 180 has been confirmed in other human-derived ovarian cell lines (Table 2).

The cell line OAW 42 was at least 540, 280 and 80 times more sensitive than D13 to DF 129, DF 229 and DF 203 respectively. DF 229 was the most potent agent, eliciting $GI_{50}$ values < 0.1 μM (Table 2). The A431 human epidermoid cell line was used as a reference line for epidermal growth factor (EGF)-related tyrosine kinase activity and gave $GI_{50}$ values ≥ 24 μM in response to challenge with DF 229 (result not shown).

A2780, A2780cis$^R$, CH1, CH1cis$^R$ and SK-OV-3-ICR cells were not sensitive to the growth-inhibitory properties of CJM 126, DF 129, DF 209 or DF 203 ($GI_{50}$ > 15 μM). The $GI_{50}$ values of DF 129 and DF 203 were lower in A2780cis$^R$ than in the cisplatin-sensitive parent cell line (2.4- to 5.5-fold respectively). This unusual

© Cancer Research Campaign 1998

**Figure 7** Effect of DF 203 (administered i.p.) on early-stage s.c. OVCAR-3 tumour xenograft growth ($n$ = 6)

property was also noted in corresponding cisplatin-sensitive and resistant lung cell lines (P Twentyman, personal communication). However, opposing these observations, $GI_{50}$ values of 0.93 and 27.3 μM were obtained when OAW 42 and D13 cells, respectively, were treated with cisplatin (data not shown). As noted, OAW 42 cells displayed the greater sensitivity to DF 129, DF 229 and DF 203 (Table 2). A2780, A2780cis$^R$, CH1, CH1cis$^R$ and SK-OV-3-ICR cell lines were challenged with DF 180, which again proved to be more potent, yielding $GI_{50}$ values between 1.25 and 4.4 μM (Table 2).

In contrast to data produced at the NCI, OVCAR-3-ICR cells were insensitive to CJM 126, DF 129, DF 209 and DF 203; $GI_{50}$ > 40 μM were obtained (Table 2). Consistent with results obtained at the NCI, IGROV1-ICR cells failed to respond to CJM 126 ($GI_{50}$ 48 μM), but were highly sensitive to DF 129 ($GI_{50}$ 23 nM) and DF 209 ($GI_{50}$ 9 nM). However, whereas DF 203 inhibited the growth of NCI IGROV1 cells ($GI_{50}$ 43 nM), the IGROV1-ICR cell line was refractive to growth inhibition and revealed a $GI_{50}$ value of 34 μM.

Colony formation of xenograft 1023 ovarian cells exposed to 0.44 μM (100 ng ml$^{-1}$) CJM 126 was inhibited by 24%, whereas 4.42 μM inhibited clonal growth by only 8% ($GI_{50}$ > 44.25 μM, Figure 6), revealing the single biphasic growth-inhibitory profile obtained after treatment with this agent. The growth of 899 and 1353 cells appeared slightly stimulated in response to CJM 126 treatment.

DF 129 induced biphasic responses in each of these three ovarian xenograft models tested in vitro. $GI_{50}$ values are given for 899 and 1023 clonal growth of 0.19 and 0.18 μM respectively (Table 2). However, $GI_{70}$ and $GI_{90}$ values were > 28.42 μM. Growth of 1353 colonies also revealed a biphasic relationship after challenge with DF 129, yet $GI_{50}$, $GI_{70}$ and $GI_{90}$ values all exceeded 28.42 μM (Figure 6).

After exposure to DF 203, 1353 cells were most responsive, yielding a biphasic clonal growth curve with $GI_{50}$ values < 0.42 μM and > 41.6 μM. $GI_{50}$ values were not reached (> 41.6 μM) after treatment of 899 and 1023 cells with DF 203 but a biphasic trend was seen in the 899 model (Figure 6).

DF 180 caused negligible inhibition of clonogenic growth at concentrations ≤ 3.31 μM. A sharp decline in viability was subsequently observed between concentrations of 3.31 μM and 33.06 μM in 1023 and 1353 tumour cells (Figure 6).

### COMPARE analysis

DF 129, DF 209, DF 229 and DF 203 were seeded in the NCI COMPARE programme: benzothiazole molecules substituted in position 3 of the phenyl ring with a halogen species or methyl group were COMPARE negative with all known classes of clinical agent. High Pearson correlation coefficients (> 0.65) revealed positive COMPARE analyses between benzothiazoles. Additionally, compound NSC 680467, 5-(4-dimethylamino-phenylazo)quinoline (Figure 1) revealed correlation coefficients > 0.68. As seed compound, NSC 680467 was COMPARE positive only with the benzothiazoles DF 129, DF 209, DF 229 and DF 203 (Table 3).

A similar profile of anti-tumour activity was demonstrated for NSC 680467 after 48-h incubation, which reflected the selectivity exhibited by substituted benzothiazoles (Table 1). Biphasic dose–response relationships were seen in sensitive IGROV1 cells ($GI_{50}$ 15 nM) and OVCAR-5 cells ($GI_{50}$ 0.68 μM), but cell numbers did not descend below the initial seeding densities. OVCAR-8 cells were refractory to growth inhibition ($GI_{50}$ > 100 μM) (Figure 2).

### In vivo assessment of ovarian anti-tumour activity

### *Hollow-fibre assays*

***DF 209*** Whereas % T/C exceeded 100% in OVCAR-3 cells implanted s.c., exposed to DF 209, i.p. implanted tumour cells responded to treatment. Inhibition was dose dependent: 27% and 65% reduction in tumour cell mass followed treatment with 100 and 150 mg kg$^{-1}$ respectively. Similarly, only the growth of OVCAR-5 cells implanted i.p. was retarded (150 mg kg$^{-1}$) (Table 4).

***DF 229*** Significant growth retardation of OVCAR-3 cultures within the peritoneum was seen (55%) after treatment with 100 mg kg$^{-1}$. However, the higher dose of 150 mg kg$^{-1}$ induced only 22% inhibition. OVCAR-3 cells implanted s.c. were inhibited by 24% and 20% by 100 and 150 mg kg$^{-1}$ DF 229 respectively (Table 4). Injection of DF 229 had negligible effect on the growth of OVCAR-5 tumour cells implanted s.c. The growth of tumour cells implanted i.p. was inhibited by 25% and 36% after treatment with 100 and 150 mg kg$^{-1}$ respectively.

***DF 203*** In the hollow-fibre in vivo model, DF 203 was the most effective of the benzothiazole anti-tumour agents tested to date. At concentrations of 6.7 and 10 mg kg$^{-1}$, significant inhibition of OVCAR-3 cell growth was observed at both tumour implantation sites: 45 ≤ % T/C ≤ 51. Inhibition of i.p. implanted OVCAR-5 cells exceeded growth inhibition at s.c. sites, with 50% and 77% inhibition of i.p. tumour growth after treatment with 6.7 and 10 mg kg$^{-1}$ DF 203 respectively (Table 4).

Growth inhibition of s.c. OVCAR-3 xenograft tumours was detected after i.p. administration of 11.2, 16.8 and 25 mg kg$^{-1}$ DF 203. However, only at the lowest dose was % T/C < 50% (Figure 7). The relationship between growth delay and DF 203 concentration was inverse. The initial mean doubling time of control tumours was 2.9 days. A maximum-tolerated dose was not

© Cancer Research Campaign 1998

reached. It was concluded that, on the treatment schedule evaluated, DF 203 did not have significant anti-tumour activity.

***DF 180*** DF 180 was essentially inactive against OVCAR-3 and OVCAR-5 cells cultured in vivo. Only at 100 mg kg$^{-1}$ was the growth of subcutaneously implanted OVCAR-5 impaired (37%); however, at the dose of 150 mg kg$^{-1}$, OVCAR-5 growth exceeded control tumour growth (Table 4).

### DISCUSSION

Novel compounds have been synthesized that demonstrated highly potent anti-tumour activity in specific ovarian (Tables 1 and 2) in addition to breast (Shi et al, 1996) cancer cell lines in vitro. An unusual feature of the dose–response relationship is the biphasic nature after 48-h DF 129, DF 209, DF 229 and DF 203 exposure. Initially, cell growth and viability decreased with increasing agent concentration (10 nM–1 μM). However, at concentrations of 10 and 100 μM, increased viability was encountered. These data are consistent with biphasic dose–response relationships observed in benzothiazole-sensitive breast cancer cell lines in which proliferation associated with DF 129, DF 209, DF 229 and DF 203 concentrations ≥ 3 μM was rapidly abolished after extended exposure periods (Bradshaw et al, 1998). The transient nature of the non-monotonic dose response was corroborated by 6-day assays performed by the NCI. Biphasic trends were abrogated in IGROV1 cultures after 6-day exposures to DF 129, DF 209, DF 229 or DF 203. However, evidence of the biphasic nature of the dose response persisted in OVCAR-5 cells.

The distinct profile of activity elicited by benzothiazoles compared with no other class of clinical agent. The biological target of these agents is unknown. DF 129, DF 209, DF 229 and DF 203 gave near identical activity fingerprints with high (> 0.65) Pearson correlation coefficients, indicating a shared biochemical mechanism(s) of action (Weinstein et al, 1997). The azoquinoline NSC 680467 was identified and subsequently evaluated in the COMPARE programme: only significant correlations were found with 3-phenyl-substituted benzothiazoles DF 129, DF 209, DF 229 and DF 203. The profile of activity of NSC 680467 in the NCI ovarian panel partly mimicked those of the benzothiazoles. As for the benzothiazoles, the mechanism of action of NSC 680467 is not known. Two MCF-7 human breast carcinoma cell lines possessing acquired resistance to CJM 126 after long-term exposure to this compound demonstrated a high degree of cross-resistance not only to benzothiazoles but also to NSC 680467, yet retained sensitivity to standard chemotherapeutic agents, such as doxorubicin, tamoxifen, mitomycin C and actinomycin D (Bradshaw et al, 1998). Therefore, a shared mechanism(s) of resistance is implied.

We have observed rapid uptake and metabolism of benzothiazoles by sensitive cells in culture: acetylated and oxidized biotransformation products have been recovered. In contrast, prostate-derived tumour cells that possess intrinsic resistance to these compounds show no net benzothiazole loss from surrounding medium and only negligible acetylation (Chua et al, manuscript in preparation).

Double substitution at position 3 and 5 of the phenyl ring with chlorine or bromine impairs biological activity. A similar consequence follows introduction of a methyl group at position 6 of the benzothiazole ring of DF 129. It is proposed that activating biotransformation processes may be hindered by such structural modifications, thus reducing compound potency.

Within the human ovarian panel, the *N*-chloroacetylated amine DF 180 failed to show any specificity in its profile of anti-tumour activity (Table 1). These observations are supported by data generated from the complete NCI tumour panel of 60 human-derived cell lines in addition to clonogenic work.

At a concentration of 10 μg ml$^{-1}$, DF 180 inhibited colony formation of 18 of 22 tumours by > 70%, whereas none of the 22 tumours, which included lung, melanoma, gastric and bladder cell lines, demonstrated this level of inhibition at 10 ng ml$^{-1}$. Compare these data with corresponding figures for DF 203: at 10 μg ml$^{-1}$ in only 2 of 22 tumours was clonogenic growth inhibition > 70%; equally at 10 ng ml$^{-1}$, in the same two tumours only, % T/C was < 30%. This illustrates the selectivity (in this case, the two responsive tumours were of breast origin) and potency of DF 203 compared with the general toxicity elicited by DF 180.

The NCI disease-oriented strategy for drug discovery is based on the hypothesis that selective activity in vitro against cancer cell lines from a specific organ may predict selective activity against corresponding tumours in vivo. Significant anti-tumour activity of DF 129, DF 203 (Shi et al, 1996) and DF 229 in breast xenograft models including MCF-7 has been demonstrated. Recently potent anti-tumour activity of 5,4′-diaminoflavone derivatives, structurally related to 2-(4-aminophenyl)benzothiazoles, has been demonstrated in MCF-7 cells in vitro and in vivo (Akama et al, 1997). We may speculate that certain pharmacological mechanisms of action may be shared. Intriguingly, two human ovarian cell lines (A2780 and OVCAR-3) were also sensitive to the growth-inhibitory properties of 5,4′-diamino-6,8,3′-trifluoroflavone.

Specificity extended to in vivo tests. The two ovarian cell lines routinely selected for implantation after culture in PVDF hollow fibres were the only cell lines whose growth was inhibited after exposure to DF 209, DF 229 and DF 203. DF 203 was significantly more toxic; the maximum-tolerated doses of DF 209, DF 229 and DF 180 were ten times that of DF 203. Indeed, DF 203, which, of the 3-substituted benzothiazoles, was the least powerful inhibitor of growth in vitro, proved the most effective agent tested in retarding the growth of OVCAR-3 and OVCAR-5 tumour cells implanted both i.p. and s.c. in hollow-fibre in vivo experiments. Evidence of the biphasic dose response observed in in vitro assays was marked in i.p. transplanted OVCAR-3 tumours exposed to DF 229 (Table 3). DF 180, as in vitro data would predict, failed to demonstrate selectivity.

The growth delay encountered after treatment of s.c. OVCAR-3 xenograft tumours with DF 203 was not significant. Delivery via the i.p. route may inefficiently reach s.c. sites. In support of this theory, growth inhibition of cells cultured in hollow fibres and implanted i.p. exceeded anti-tumour activity at s.c. sites, after exposure to DF 209 and DF 229. The inverse relationship observed between DF 203 concentration and % T/C appears to reflect in vitro observations. Metabolic routes of anti-tumour benzothiazoles in vivo and in vitro are being investigated.

Finally, the promising anti-tumour potential of these novel benzothiazole molecules, ease of syntheses (Stevens et al 1995; Shi et al, 1996), high yields, simple structures and compound stability appear to justify further preclinical development.

### ABBREVIATIONS

NCI, National Cancer Institute; MTT, 3-(4,5-dimethylthiazol-2-yl)-2,5-diphenyltetrazolium bromide; $GI_{50}$, $GI_{70}$ and $GI_{90}$ concentrations at which 50%, 70% and 90% inhibition were encountered.

© Cancer Research Campaign 1998

### ACKNOWLEDGEMENTS

This paper is part 6 of the series 'Antitumour benzothiazoles'. This work was supported by the Cancer Research Campaign, UK. The authors would like to acknowledge helpful discussions with members of the Screening and Pharmacology Group of the EORTC.

The manuscript is dedicated to Dr KD Paull.

### REFERENCES

Abu Sinna G, Beackman G, Lundgren E and Roos G (1979) Characterisation of two new ovarian carcinoma cell lines. *Gynecol Oncol* **7**: 267–280

Akama T, Ishida H, Shida Y, Kimura U, Gomi K, Saito H, Fuse E, Kobayashi N, Yoda N and Kasai M (1997) Design and synthesis of potent 5,4′-diaminoflavone derivatives based on metabolic considerations. *J Med Chem* **40**: 1894–1900

Boyd MR (1989) Status of the NCI preclinical antitumor drug discovery screen. In *Cancer: Principles and Practice of Oncology Updates*, Vol. 3, DeVita VT, Hellman S and Rosenberg SA. (eds), JP Lippincott: Philadelphia, pp. 1–12

Bradshaw TD, Wrigley S, Shi D-F, Schultz RJ, Paull KD and Stevens MFG (1998) 2-(4-Aminophenyl)benzothiazoles: novel agents with selective profiles of in vitro anti-tumour activity. *Br J Cancer* **77**: 745–752

Hamburger AW and Salmon SE (1977) Primary bioassay of human tumor stem cells. *Science* **197**: 461–463

Monks A, Scudiero D, Skehan P, Shoemaker R, Paull K, Vistica D, Hose C, Langley J, Cronise P, Vaigro-Wolfe A, Gray-Goodrich M, Campbell H and Boyd MR (1991) Feasibility of a high-flux anticancer drug screen utilizing a diverse panel of human tumour cell lines in culture. *J Natl Cancer Inst* **83**: 757–766

Monks A, Scudiero DA, Johnson GJ, Paull KD and Sausville EA (1997) The NCI anti-cancer drug screen: a smart screen to identify effectors of novel targets. *Anti-Cancer Drug Design* **12**: 533–541

Shi D-F, Bradshaw TD, Wrigley S, McCall CJ, Lelieveld P, Fichtner I and Stevens MFG (1996) Antitumour benzothiazoles. 3. Synthesis of 2-(4-aminophenyl)benzothiazoles and evaluation of their activities against breast cancer cell lines *in vitro* and *in vivo*. *J Med Chem* **39**: 3375–3384

Stevens MFG, McCall CJ, Lelieveld P, Alexander P, Richter A and Davies DE (1994) Structural studies on bioactive compounds. 23. Synthesis of polyhydroxylated 2-phenylbenzothiazoles and a comparison of their cytotoxicities and pharmacological properties with genistein and quercetin. *J Med Chem* **37**: 1689–1695

Stevens MFG, McCall CJ and Lelieveld P (1995) Benzazole compounds for use in therapy. International Application Number WO 95/06469; Stevens MFG, Shi D-F, Bradshaw TD and Wrigley S. Benzazole compound. Br. Patent, 9503946.7

Weinstein JR, Myers TG, O'Connor PM, Friend SH, Fornace AJF, Kohn KW, Fojo T, Bates SE, Rubinstein LV, Anderson NL, Buolamwini JK, van Osdol WW, Monks AP, Scudiero DA, Sausville EA, Zaharevitz DW, Bunow B, Viswanadhan VN, Johnson GS, Wittes RE and Paull KD (1997) An information-intensive approach to the molecular pharmacology of cancer. *Science* **275**: 343–349

Wilson AP (1984) Characterisation of a cell line derived from the ascites of a patient with papillary serous cystadenocarcinoma of the ovary. *J Natl Cancer Inst* **72**: 513–521

Yates PC, McCall CJ and Stevens MFG (1991) Structural studies on benzothiazoles. Crystal and molecular structure of 5,6-dimethoxy-2-(4-methoxyphenyl)-benzothiazole and molecular orbital calculations on related compounds. *Tetrahedron* **47**: 6493–6502

© Cancer Research Campaign 1998